# High Adhesion and Increased Cell Death Contribute to Strong Biofilm Formation in *Klebsiella pneumoniae*

**DOI:** 10.3390/pathogens8040277

**Published:** 2019-12-01

**Authors:** Siddhi Desai, Kinjal Sanghrajka, Devarshi Gajjar

**Affiliations:** Department of Microbiology and Biotechnology Centre, Faculty of Science, The Maharaja Sayajirao University of Baroda, Vadodara-, Gujarat 390002, India; sid.desai21@gmail.com (S.D.); sanghrajkakinjal03@gmail.com (K.S.)

**Keywords:** *Klebsiella*, biofilms, adhesion, eDNA, cell death, catheter

## Abstract

*Klebsiella pneumoniae* (*Kp*), is a frequent cause of hospital and community-acquired infections and WHO had declared it as a “priority pathogen”. Biofilm is a major virulence factor of *Kp* and yet the mechanism of strong biofilm formation in *Kp* is unclear. A key objective of the present study is to investigate the differences between strong and weak biofilms formed by clinical isolates of *Kp* on various catheters and in different media conditions and to identify constituents contributing to strong biofilm formation. Quantification of matrix components (extracellular DNA (eDNA), protein, exopolysaccharides (EPS), and bacterial cells), confocal laser scanning microscopy (CLSM), field emission gun scanning electron microscopy (FEG-SEM) and flow-cytometry analysis were performed to compare strong and weak biofilm matrix. Our results suggest increased biofilm formation on latex catheters compared to silicone and silicone-coated latex catheters. Higher amounts of eDNA, protein, EPS, and dead cells were observed in the strong biofilm of *Kp*. High adhesion capacity and cell death seem to play a major role in formation of strong *Kp* biofilms. The enhanced eDNA, EPS, and protein in the biofilm matrix appear as a consequence of increased cell death.

## 1. Introduction

*Kp* is the most common causative agent of nosocomial Gram-negative bacteremia and urinary tract infections (UTI) after *E. coli* [1,2]. Among *Klebsiella* spp., *Kp* is a prominent etiological agent of nosocomial and community acquired infections and has emerged as an “urgent threat” to public health due to multidrug resistance. Biofilms are a major issue in healthcare and are reported to be involved in 65% of bacterial infections, allowing cells to persist and leading to increased antibiotic resistance [3]. An epidemic of drug resistant *Kp* is reported due to the dissemination of KPC-3 carrying *Kp* [4,5]. Further, catheter-associated urinary tract infections (CAUTIs) by *Kp* represent one of the most common hospital-acquired infections (HAIs) leading to increased patient morbidity [6]. Bacterial biofilm formation on the interior and exterior surfaces of the catheter has been identified as the most important cause of CAUTIs [7]. Biofilm is an aggregate of microorganisms attached to an inert or living surface by a self-produced exo-polymeric matrix, which include polysaccharides, proteins, and extracellular DNA (eDNA) [8]. Biofilms inhibit effective antibiotic penetration, reduce the bacterial growth rate, lead to the development of persister cells, and facilitate genetic exchange [9,10]. Hence, a detailed understanding of the biofilm may help in developing strategies to combat biofilm formation.

Recently, several studies report the association of antibiotic resistance with biofilm formation in clinical isolates of *Klebsiella* spp. [11,12,13,14]. Moreover, the role of fimbriae in adhesion and biofilm formation by *Klebsiella* is also well documented [15,16]. However, studies on characterization and quantification of *Kp* biofilms are lacking. The present work is undertaken to explore the differences between strong and weak biofilms formed by clinical isolates of *Kp* on various catheters and in different media conditions and differences in their matrix components.

## 2. Results

### 2.1. Biofilm Formation by Uropathogenic Kp

**Figure 1:**
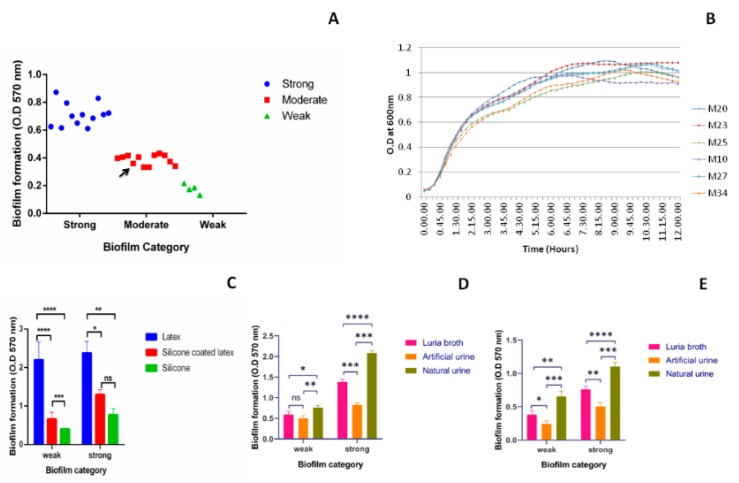
Biofilm formation by uropathogenic *Kp*: Biofilm formation by uropathogenic *Kp* isolates (*n* = 28) using crystal violet assay on 96-well plate and different catheter materials in the presence of different media. Black arrow indicates biofilm formation by microbial type culture collection (MTCC) *Kp* 39. (**A**) Quantification and categorization of biofilm producer isolates. (**B**) Growth curve of weak (M-20,23,25) and strong (M-10,27,34) biofilm producers at 15 min interval till 12 h. (**C**) Biofilm formed by strong (M-10,27,34) and weak (M-20,23,25) biofilm producers on latex, silicone-coated latex and silicone catheters. Biofilm formed in the presence of Luria-Bertani (LB) broth, artificial urine, and natural urine on (**D**) silicone-coated latex and (**E**) silicone catheters. Statistical analysis was performed by the unpaired *t*-test using Prism 8 GraphPad. * *p* < 0.05; ** *p* < 0.01, *** *p* < 0.001, **** *p* < 0.0001, ns *p* > 0.05.

Biofilm formation by all the clinical isolates (*n* = 28) was studied using crystal violet assay in a 96-well polystyrene plate and categorized as per their biofilm forming ability (Figure 1A). Majority of the isolates were able to form a strong or moderate type of biofilm. A non-pathogenic microbial type culture collection (MTCC) strain 39 of *Kp* also formed moderate level of biofilm. Among all the collected isolates 43%, 43%, and 14% were strong, moderate, and weak biofilm producers, respectively (Figure 1A). From 28 biofilm producer isolates of different categories, three weak, M-20,23, and 25 and three strong (M-10,27, and 34) biofilm producers were selected randomly for further study. The average growth rate of these selected isolates measured was 0.841 ± 0.03/h (Figure 1B). MLST types of M-20,23,25,10,27, and 34 are ST2943, ST10, ST1087, ST2491, ST1715, and ST38, respectively. Biofilm formation by these six isolates on various catheters (Figure 1C) in presence of different media (Figure 1D,E) was investigated. In case of weak biofilm, significant difference in biofilm formation between latex and silicone coated latex, as well as silicone catheters was observed (*p* < 0.0001) (Figure 1C). The difference in biofilm formation between silicone coated latex and silicone catheters was also significant with *p* < 0.001 in weak biofilms. In case of strong biofilm, significant difference between latex and silicone (*p* < 0.01); latex and silicone-coated latex (*p* < 0.05) was observed. No significant difference between silicone-coated latex and silicone was observed in case of strong biofilm. When biofilm formation was studied on two types of catheters, in the presence of different media, significant increase in biofilm formation was observed in case of natural urine as compared to Luria-Bertani (LB) broth and artificial urine on silicone-coated latex catheter (Figure 1D) and silicone catheter (Figure 1E) (*p*-value shown in figures). Hence, the biofilm formation was lowest on silicone catheters followed by silicone-coated latex and latex catheters. In the presence of different media, the biofilm formation was highest in natural urine followed by LB and artificial urine.

### 2.2. Components of Strong and Weak Biofilm Matrix

**Figure 2:**
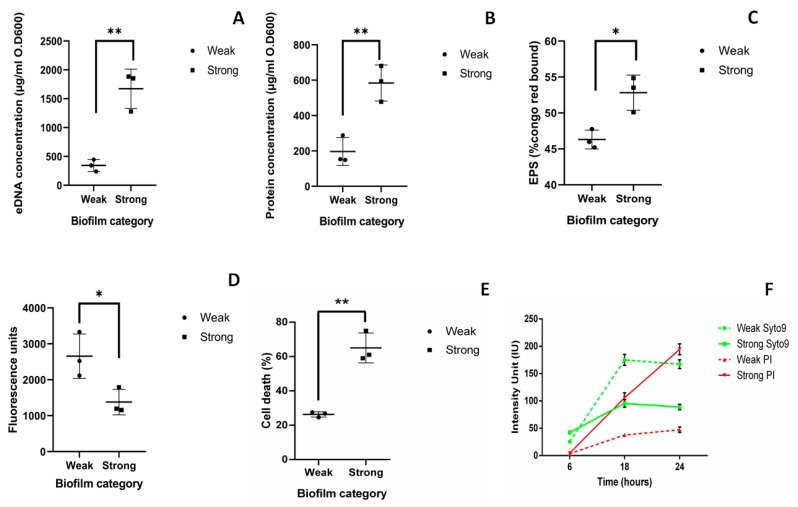
Components of strong and weak biofilm matrix: Quantification of components of biofilm matrix was performed from biofilms grown on 12-well polystyrene plate after 48 h and normalized with the optical density (OD) at 600 nm. Quantification was done from biofilms formed by three weak (M-20,23,25) and three strong (M-10,27,34) biofilm producers. (**A**) eDNA was extracted using the phenol-chloroform method and quantified using nanodrop. (**B**) Amount of protein present in biofilm matrix was quantified using the Bradford method. (**C**) Total exopolysaccharides (EPS) present in the biofilm matrix was quantified in terms of percentage congo red bound using Congo red method. (**D**) The measure of live cells in biofilm was obtained using a resazurin assay in terms of average fluorescence unit. (**E**) Flow-cytometry analysis of live dead assay using BacLight kit (syto9 and propidium iodide (PI)). (**F**) Intensity of live cells stained with syto9 (green) and dead cells stained with PI (red) measured at 6, 18, and 24 h in weak (dotted line) and strong (solid line) biofilms by time bound live dead assay. Statistical analysis was performed by the unpaired *t*-test using Prism GraphPad. *, *p* < 0.05; **, *p* < 0.01.

The average amount of eDNA quantified from weak biofilm matrix (344.5 µg/OD_600_) was lower when compared to eDNA from strong biofilm matrix (1673 µg/OD_600_), which was significantly higher (*p* < 0.01) (Figure 2A). The average amount of extracellular protein present in weak and strong biofilm matrix was 197.1 and 584.4 µg/OD_600,_ respectively (Figure 2B). Exopolysaccharides (EPS) obtained in weak and strong biofilm matrix was 46.31% and 52.38%, respectively (Figure 2C). The measure of live cells in biofilm was obtained using resazurin assay. Average fluorescence units (FU) obtained in weak and strong biofilms were 2658 and 1381 FU, respectively (Figure 2D). Significantly less number of live cells were found in strong biofilm than weak biofilm (*p* < 0.05). Previously, it has been reported that the live cells measured by resazurin assay and estimated CFU present in biofilm show negligible amount of variation. Hence, we have performed resazurin assay for quantification of live and dead cells present in the biofilm [17].

The number of dead cells present in weak and strong biofilm was evaluated using flow- cytometry analysis after 48 h. 23% of dead cells (Propidium iodide (PI) positive cells) were observed in weak biofilm as compared to 65% in strong biofilm. This indicates that more number of dead cells were present in strong biofilm than weak biofilm with *p* < 0.01 (Figure 2E).

Time bound live dead assay was done at 6, 18, and 24 h to see the live dead ratio in weak and strong biofilms. In case of strong biofilm, cell death (intensity of PI) was observed to be increased at 18 h (105 ± 9 IU) than at 6 h (4.4 ± 0.5 IU) and maximum intensity of PI was measured at 24 h (194 ± 10 IU). Whereas, in case of weak biofilm cell death (intensity of PI) was high at 18 h (37 ± 1 IU) than at 6 h (3.4 ± 0.3). However, in 24 h, intensity of PI was significantly lesser (47 ± 5 IU) compared to that of the strong biofilm (194 ± 10 IU). (Figure 2F). Though the growth rate of all the isolates are similar, cell death was found to be increased with the time only in strong biofilms.

To summarize, significantly higher amount of eDNA (*p* < 0.001), protein (*p* < 0.001), EPS (*p* < 0.05), and dead cells (*p* < 0.05) were observed in strong biofilms than in weak biofilms.

#### Inhibition and Addition Assay

**Figure 3:**
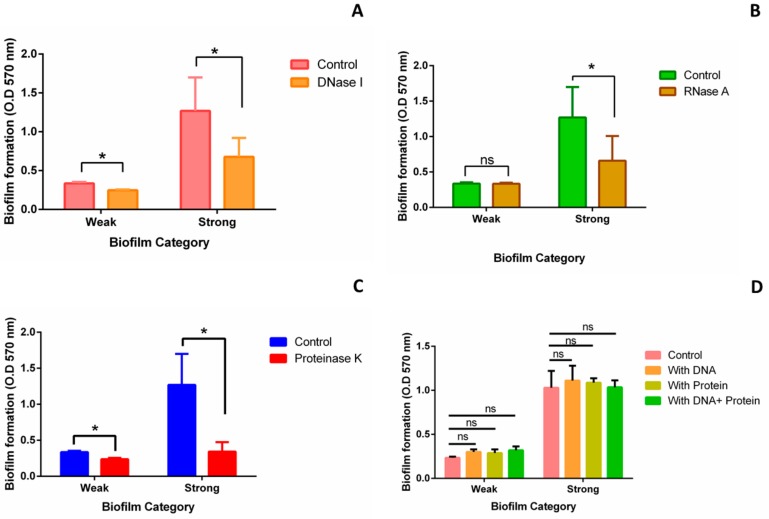
Inhibition and addition assay: Inhibition assay was performed to validate the role of eDNA, RNA (Ribonucleic acid), and protein in biofilm formation. Biofilms were allowed to form for 24 h in a 96 well-plate. Respective enzymes were added after 24 h and further incubated for 24 h at 37 °C. Crystal violet assay was performed to quantify the biofilm. Biofilm formed without the treatment of enzymes was used as control. Quantification of weak and strong biofilms after the treatment of (**A**) DNase I (**B**) RNase A, and (**C**) Proteinase-K. Concentration of all three enzymes used for the treatment was 100 μg/mL. For addition assay, *Kp* cell extracted DNA and proteins were added separately and both together at 0 h and biofilm was allowed to form for 24 h at 37 °C. (**D**) Quantification of weak and strong biofilm formed after 24 h in the presence of additional *Kp* cell extracted DNA and protein. Concentration of DNA and protein used for the treatment was 3 μg/mL. Statistical analysis was performed by the unpaired *t*-test using Prism GraphPad. *, *p* < 0.05.

To further validate the role of different matrix components in biofilm formation, we performed inhibition and addition assays. In case of strong biofilm, significant reduction in biofilm was observed after treating the biofilm with DNase I (46.62%) (Figure 3A), RNase A (48.12%) (Figure 3B), and Proteinase K (72.9%) (Figure 3C). In case of weak biofilm, biofilm was reduced by 26.19%, 0.1%, and 29.4% upon DNase I (Figure 3A), RNase A (Figure 3B), and Proteinase K (Figure 3C) treatment, respectively. However, exogenous addition of *Kp* cell extracted DNA and protein to both weak and strong biofilms did not show any significant change in biofilm formation (Figure 3D).

### 2.3. Microscopy of Weak and Strong Biofilm

**Figure 4:**
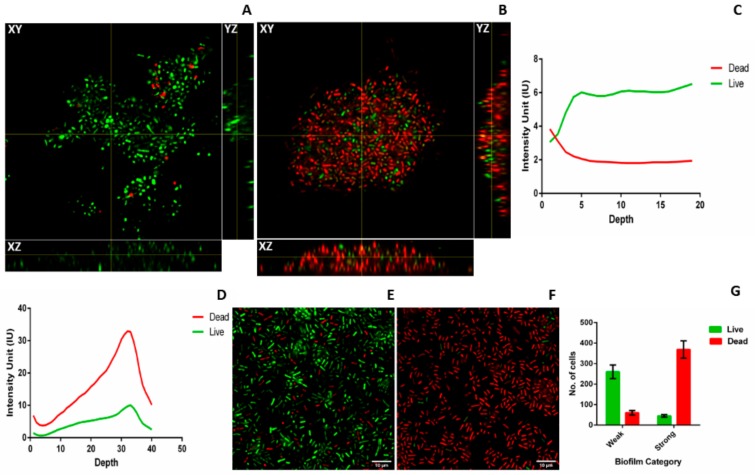
Confocal laser scanning microscopy (CLSM) of weak and strong biofilms: Biofilm formed by three strong and three weak biofilm producers on coverslip after 48 h were subjected to CLSM. Representative orthogonal view of the Z-stack (**A**) weak and (**B**) strong biofilms are shown with XY, YZ, and XZ planes. Intensity of syto9 (green) and PI (red) was measured across the depth of the Z-stack and live dead ratio was estimated based on intensity units (IU) for both weak(**C**) and strong (**D**) biofilm. Representative tile images of weak (**E**) and strong (**F**) biofilm shows the distribution of live (green) and dead (red) cells in the biofilm matrix. Array of multiple images is presented as a single tile image. (**G**) Quantification of number of live and dead cells present in weak and strong biofilm. Images were analyzed using ImageJ2 and Zen Zeiss microscope software.

To characterize the weak and strong biofilms, confocal laser scanning microscopy (CLSM), light microscopy, and field emission gun scanning electron microscopy (FEG-SEM) were performed for three weak (M-20,23,25) and three strong (M-10,27,34) isolates. To visualize 3D structure of live and dead cells embedded inside the biofilm matrix and to further evaluate the huge amount of cell death observed in strong biofilms, CLSM was performed. Distinct differences in the biofilm structure and thickness were observed between strong and weak biofilms formed by all six biofilm producers in CLSM (Figure 4). Orthogonal views of weak and strong biofilms show differences in thickness and arrangement of live and dead cells in the biofilm matrix. Weak biofilm (Figure 4A) was observed to be sparsely packed with more numbers of live cells whereas strong biofilm was densely populated with more numbers of dead cells compared to live cells (Figure 4B). YZ and XZ planes of Figure 4A,B also give information about the difference in the thickness of weak and strong biofilms. Figure 4C,D indicates the measure of live and dead cells based on the intensity units. It was observed that in weak biofilm, the number of live cells increase and dead cells decrease with the increase in the depth (Figure 4C). In strong biofilms, the number of dead cells increase and live cells decrease with increase in the depth (Figure 4D). It was also observed that the thickness of weak biofilm was observed to be only 19 slices thick whereas, strong biofilm was observed to be expanded up to 40 slices with the uniform slice interval of 0.36 µm. This also indicates a significant difference in the thickness of weak (7 ± 2 µm) and strong biofilms (14 ± 1 µm). Tile image of weak biofilm showed the presence of less number of dead cells and more number of live cells (Figure 4E). On other hand, tile image of strong biofilm showed a large number of dead cells and very less number of live cells (Figure 4F). The number of live and dead cells in weak biofilm were 260 ± 33 and 60 ± 11, respectively in the area of 1000 × 1000 pixel (region of interest (ROI)) of the tile image. The number of live and dead cells in strong biofilm are 45 ± 6 and 369 ± 42 cells, respectively (Figure 4G). The difference in cell death between FACS and CLSM is due to the difference in the assays. Interestingly, 3D structure of strong biofilm showed pockets of live cells embedded within the thick layers of dead cells (Supplementary Videos).

**Figure 5:**
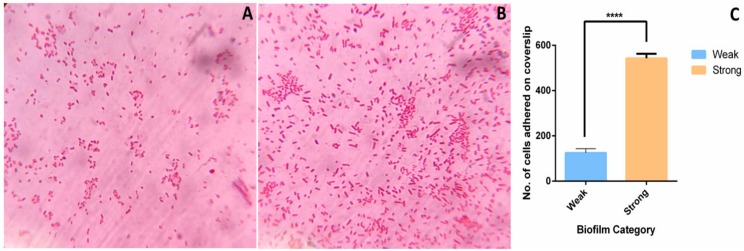
Adhesion assay of weak and strong biofilms: Adhesion assay was performed to evaluate the adhesion ability of weak and strong biofilm producers. For adhesion assay, biofilm was allowed to form on a coverslip for 4 h at 37 °C and then subjected to gram staining followed by light microscopy. Representative light microscopy images of (**A**) weak and (**B**) strong biofilm producers adhered to the coverslip after 4 h are shown. (**C**) Average number of cells adhered in weak and strong biofilms after 4 h of cell adhesion assay. ImageJ2 was used to analyze the images. ****, *p* < 0.0001.

To study the differences in adhesion capacity, three weak and three strong biofilm producers were subjected to cell adhesion assay followed by light microscopy. Very few Gram-negative rods in light microscopy were observed to be adhered in weak biofilm producers (Figure 5A). Conversely, large number of cells were observed to be adhered in strong biofilm (Figure 5B) during early biofilm stage (4 h). Number of cells adhered to the coverslip after 4 h of biofilm formation were 125 ± 18 and 542 ± 20 in weak and strong biofilms (Figure 5C). This indicates that adhesion capacity of strong biofilm producers is higher than the weak biofilm producers.

**Figure 6:**
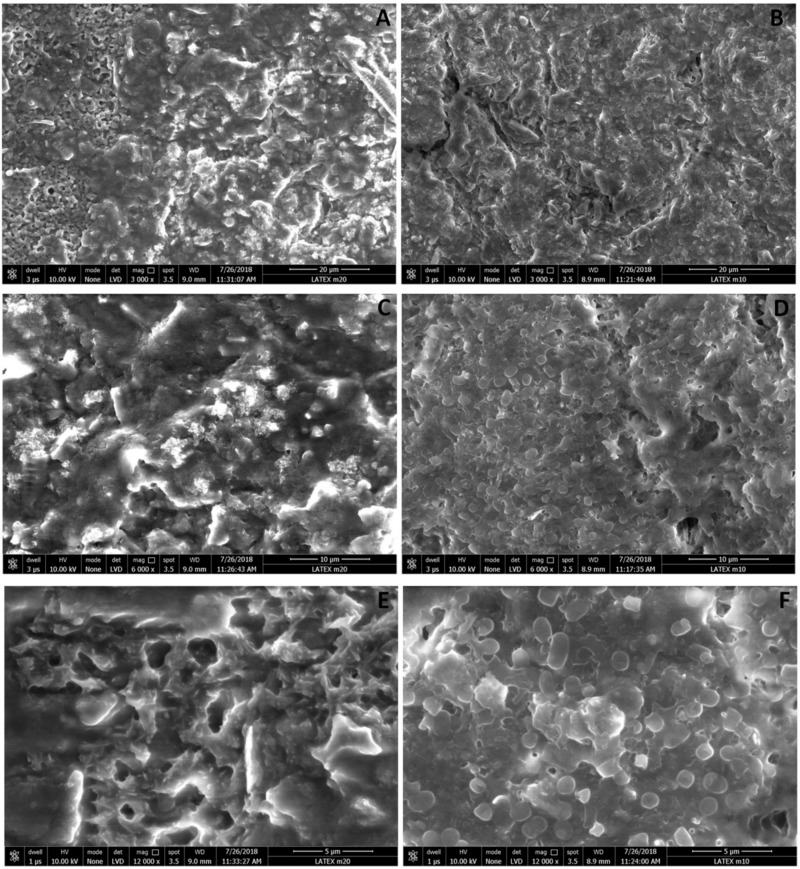
FEG-SEM of weak and strong biofilms: FEG-SEM of weak and strong biofilms formed on 13 mm long piece of silicone-coated latex catheter. SEM observation was performed at room temperature in environmental mode for weak biofilm (**A**,**C**,**E**) and strong biofilm (**B**,**D**,**F**) and observed at 3000×, 6000×, and 12,000× magnification, respectively. Scale bars represent 20 (**A**,**B**); 10 (**C**,**D**), and 5 µm (**E**,**F**).

FEG-SEM was performed to investigate the differences in the structure of weak and strong biofilms formed on silicone-coated latex catheters. FEG-SEM micrographs of weak biofilms showed very less number of cells embedded in cloud like EPS. It also suggests the presence of micro-channel like structures in the network of exo-polymeric matrix (Figure 6A,C,E). On other hand, the strong biofilm micrographs showed higher number of interconnected cells embedded in densely populated and abundant extracellular matrix (Figure 6B,D,F).

## 3. Discussion

Despite biofilm formation by *Kp* has been extensively studied [18,19,20,21,22], the mechanism of strong biofilm formation in *Kp* is underexplored. Moreover, how strong and weak *Kp* biofilm differ from each other is unclear. The present study extends the knowledge about constituents contributing to strong *Kp* biofilms. In this study, clinical isolates from UTIs showed varying levels of biofilm formation in the CV assay. Furthermore, the biofilm index (Biofilm index = OD570_(CV assay)_/OD 600_(culture)_) and OD of bacterial culture after 24 h of biofilm formation, before washing of unbound cells was measured to validate that the difference in biofilm formation is not because of difference in the growth rate of the bacteria [23]. Further, no difference in growth rate was found in strong and weak biofilm producers (Figure 1B). Variation in the rate of biofilm formation, stages of biofilm formation, and structural differences between strong and weak biofilm have been reported previously [24].

Results of biofilm formation on latex, silicone-coated latex and silicone catheters show high, moderate, and low biofilm formation, respectively. Another important observation was that *Kp* isolates with weak biofilm forming capacity formed a strong biofilm on latex urinary catheters. The issue with latex is its cytotoxicity in addition to increased biofilm formation. Hence, latex catheters are now coated with silicone elastomer to reduce this risk. Many modern catheters are made entirely of silicone and hydrophilic coatings, which are used to provide a slippery surface to reduce attachment [25]. Silicone catheters are not only hypoallergenic, but they also have shown reduced biofilm formation compared to latex [26,27]. Lee et al. have reported that the rough surface of latex catheters make the microbial attachment easy and an additional amount of biofilm formation occurs, whereas smooth surface and less hydrophobicity of silicone catheters are responsible for reduced biofilm formation [28]. Our results corroborate with these findings in favor of silicone catheters to be preferred over latex with respect to biofilm formation. However, the cost of latex catheter is five times lower than silicone catheters and hence, latex is coated with silicone and is the preferred choice in clinical settings of most developing countries. To increase the resemblance with the clinical scenario in developing countries, quantification of biofilm on silicone-coated latex catheters (widely used catheters) in the presence of urine was performed. Composition of growth medium and substratum are known to have influence on the production of extracellular components and biofilm density [29] and our findings further validate these reports.

The second aim of the study was to quantify and compare the components of weak and strong biofilms. Results in the present study show high eDNA, protein, EPS, cell adhesion, and unusual cell death in strong biofilms. Detailed studies on *Pseudomonas aeruginosa* biofilms have shown that eDNA forms complexes with exopolysaccharides [30] and crosslinks with proteins [31,32]. This increases mechanical strength and adhesion capacity of the bacteria by acid-base interaction with the surfaces [33]. Moreover, polysaccharides, proteins, and DNA allow the initial steps in the colonization and temporary immobilization of bacterial cells to the surfaces [34,35]. Presence and importance of eDNA in *Kp* biofilms was shown by giving DNase treatment which led to reduced biofilm formation [36]. Harmsen et al. have also done the experiments with the addition of salmon sperm DNA, genomic DNA, and DNase I in biofilm formation of *Listeria monocytogenes*. Significantly reduced biofilm was observed upon the treatment with DNase I. However, no significant increase was reported in case of addition of genomic DNA or salmon sperm DNA. They also concluded that the size of the intercellular molecules matters and reducing the size of these molecules and addition of the single components do not increase adhesion or biofilm formation [37]. Our results with *Kp* biofilms corroborate with these reports, that in *Kp* biofilms, significant reduction was observed upon treatment with DNase I, as well as no significant increase was seen upon the addition of *Kp* genomic DNA. In addition to DNase I, no significant effect in the presence of RNase A and Proteinase K was reported in case of *Listeria monocytogenes* [37]. However, we have observed significant reduction in both weak and strong biofilms upon treatment with Proteinase K. Moreover, biofilm was significantly reduced after treatment of RNaseA in case of strong biofilms.

Preliminary observations of increased cell death in strong biofilms came from the results of the resazurin assay. Further, FACS and CLSM were performed to confirm these observations. The difference between % cell death measured by FACS and CLSM is due to difference in the experimental procedure of live dead assay. Cell death has been reported to be crucial in case of *S. aureus* and *P. aeruginosa* biofilms [38]. The multicellular structure of biofilm provides a selective pressure for programmed cell death which eliminates damaged cells and enhances nutrient availability for the healthy cells in the biofilm matrix [39]. Cell death is caused by self-destruction of individual cells and lysis of dead bacteria releases genomic DNA [40]. Except programmed cell death, several other mechanisms are reported for eDNA release such as membrane vesicle formation [41], prophage-mediated cell death in *Pseudomonas aeruginosa* [42], and specialized secretion in *Neisseria gonorrhoeae* [43]. However, in *Kp* the mechanism for the release of eDNA is unclear. We hypothesize that cell death could be the cause of increased eDNA, protein, and EPS in strong biofilms. The arrangement observed in the 3D structure of confocal microscopy indicates that dead cells could act as physical barriers protecting the live cells inside the matrix. Cell death during biofilm formation is an ordered and well-regulated process [38]. Though cell death during biofilm formation is one of the least understood processes; three models have been proposed that lead to cell death [44]. 1. Bacteria at the base of the microcolony die as nutrients are unable to reach the innermost layer of the biofilm. 2. Bacteria even in the outer layers of the biofilm die and metabolism of the neighboring bacteria seem to contribute to death. 3. Bacteria die due to accumulation of damage at the top of the microcolony. Any of the above-mentioned theories could be playing a role simultaneously during *Kp* biofilm formation. Thus, what causes such increased cell death warrants further investigation.

In addition to all these factors, fimbriae is also reported as one of the factors associated with biofilm formation. *fim* gene cluster encoding type 1 fimbriae and *mrk* gene cluster encoding type-3 fimbriae were found to be present in whole genome of all six isolates (both strong and weak biofilm producers) (data not shown). Previously it is also reported that both, type 1 and type 3 fimbria-encoding operon is found in and expressed by almost all *Kp* isolates in biofilm state and both, type 1 and type 3 fimbriae play a role in the formation of biofilm [16,45]. Hence, absence of fimbriae can prevent biofilm formation, but it may not be solely responsible for either strong or weak biofilm. Further, we have no evidence to indicate that disease severity correlates with strong or weak biofilm formers, we do not claim the “strong” to be associated with disease severity or its ability to withstand the biophysical stress.

Bandeira et al. had studied the ability of various *Kp* strains to assemble biofilms and relative area occupied by bacteria and extracellular polymeric substances on cell culture plates using SEM. They have categorized the strains into most and the least efficient, as well as intermediately efficient biofilm assembler [8]. Singla et al. have shown 3D structure of *Kp* biofilms with enhanced exopolysaccharide production and water channel formation [46]. Here, we report SEM of weak and strong *Kp* biofilms grown on silicone-coated latex catheters with more number of bacteria and increased EPS in strong biofilm compared to weak.

To summarize, we found heterogeneity in biofilm formation by clinical isolates, increased biofilms on latex compared to silicone catheters and increased biofilms in the presence of natural urine. Latex catheters used in healthcare settings of developing countries due to its cost effectiveness seem to contribute to the high prevalence of biofilm associated infections. High eDNA, protein, EPS, cell adhesion, and unusual cell death were found to be associated with the strong biofilms. It is evident that increased eDNA, protein, and RNA in strong biofilm matrix is a consequence of cell death.

## 4. Materials and Methods

### 4.1. Bacterial Isolates and Growth Conditions

Clinical isolates (*n* = 28) of *Klebsiella* spp. were collected from pathology labs in Surat and Vapi, South Gujarat, India. All collected isolates are from patients suffering from urinary tract infection (UTI). Identification of all isolates was done by biochemical tests, Vitek-2 system (bioMérieux, Marcy-l′Étoile, France) and 16s *rRNA* gene sequencing. Species level identification was done using 16s rRNA and all collected isolates were identified as *Klebsiella pneumoniae*. Isolates were cultured on MacConkey agar for routine maintenance and storage.

### 4.2. Quantification of Biofilm Formation

Crystal violet assay was performed to quantify biofilm formation by all 28 clinical isolates of *Kp* and further categorized into strong, moderate, or weak biofilm producers using statistical analysis described by Stepanović et al. 2004 [47]. Briefly, 25 µL of overnight grown culture (O.D. at 600 nm ~ 0.3) was added to 225 µL of sterile LB in a sterile 96-well flat bottom microtiter plate (Laxbro Bio-Medical Aids pvt. Ltd., Pune, India) and incubated at 37 °C for 24 h. The assay was performed in triplicates. Only LB without bacterial culture was used as negative control for biofilm formation and MTCC K. pneumoniae 39 strain was taken as a standard strain from Microbial Type Culture Collection (MTCC). After 24 h, the adhered biofilm was fixed by adding 250 µL of methanol for 15 min. The biofilm formed was then stained by 250 µL of 0.5% crystal violet solution for 15 min. The excess stain was washed away by flushing the wells with 0.8% saline twice and then allowed to be air dried. The stain attached to adherent layers was re-solubilized in 250 µL of 33% acetic acid for 15 min. The optical density (OD) of the solution was measured at 570 nm using a microtiter plate reader (Multiskan Go, Thermo Fisher Scientific, Waltham, MA, USA). The isolates were categorized into weak, moderate, or strong biofilm producers based on the cut-off OD Cut-off OD (ODc) is defined as three standard deviations above mean OD of negative control (at 570 nm). Isolates were classified as follows: OD ≤ ODc = no biofilm producer, O.Dc < O.D. ≤ (2 × ODc) = weak biofilm producer, 2ODc < OD ≤ (4 × ODc) = moderate biofilm producer, (4 × ODc) < OD = strong biofilm producer [47]. Moreover, the biofilm index was calculated for all 28 isolates using this formula. Biofilm index = OD570_(CV assay)_/OD 600_(culture)_ [23].

### 4.3. Growth Curve

Growth curve assay was performed for all six isolates using synergy HTmicroplate reader (BioTek instruments, Winooski, VT, USA). 250 µL of overnight grown culture (OD at 600 nm ~ 0.3) was added to 1 mL of fresh LB and incubated at 37 °C until the OD at 600 nm reaches 0.05. 100 µL of all six isolates (OD at 600 nm ~ 0.05) was inoculated in a sterile 96-well flat bottom microtiter plate and incubated at 37 °C for 24 h in continuous shaking conditions in an automated microplate reader. OD at 600 nm was measured constantly at an interval of 15 min for 12 h until the cultures reach to the stationary phase and growth curve was plotted. The growth rate and generation time for each isolate was calculated from the graph.

### 4.4. Quantification of Biofilm on Catheters

Biofilm formed on three types of catheters (latex, silicone-coated latex, and silicone) was quantified by a modified crystal violet assay. 13 mm long piece of each type of catheter was cut vertically and fixed at the base of a 24-well plate (Laxbro Bio-Medical Aids Pvt. Ltd., Pune, India). 1 mL of 1:10 diluted culture (OD at 600 nm ~ 0.3) was inoculated in wells containing catheter piece and incubated at 37 °C for 24 h. After 24 h, unbound cells were washed with water. The piece of catheter was then transferred to a fresh 24-well plate to avoid the evaluation of the biofilm formed at the bottom of the previous plate. Bound biofilm was fixed with 1 mL methanol and then stained with 1 mL 0.5% crystal violet. The excess stain was washed with 0.8% saline, the bound stain was re-solubilized in 1 mL 33% acetic acid and its OD was measured at 570 nm. Biofilm formed in the presence of sterile LB, sterile artificial urine (2.43% urea, 1% NaCl, 0.6% KCl, 0.64% Na2HPO4, 0.05 mg/mL albumin, pH 5–7) [48], and natural urine was quantified using the same assay. The assay was performed in triplicates. Only LB, sterile artificial urine and natural urine without bacterial culture was used as negative control for biofilm formation in each type of medium.

### 4.5. Quantification of Components of Biofilm Matrix

eDNA, protein, EPS, and cells present in the matrix of weak and strong biofilms were quantified and normalized with OD 600 [49,50]. 2 mL of 1:10 diluted culture (OD at 600 nm ~ 0.3) was inoculated in a 24-well plate and incubated at 37 °C for 48 h. The wells were decanted after 48 h and the biofilm was re-solubilized in 1 mL of 0.8% saline. The biofilm formed in 6-wells were pooled, SDS was added to final concentration of 0.01% and incubated at room temperature for 4 h at 150 rpm. Cell debris were removed by centrifugation at 5000× *g* for 5 min and the supernatant was passed through 0.2 µm cellulose acetate filter (Sartorious stedim Biotech Pvt. Ltd., Göttingen, Germany) and the filtered solution was used for eDNA and protein quantification. 1.5 mL of the pooled sample (before SDS treatment) was used for EPS quantification and live dead assay using flow cytometer.

#### 4.5.1. eDNA Quantification

The phenol-chloroform method was used for eDNA extraction and quantified using nanodrop (Thermo Fisher Scientific, Waltham, MA, USA) [49]. 500 µL of filtered solution was subjected to phenol-chloroform method to extract the extracellular DNA. Equal volumes of henol, chloroform, and isoamyl alcohol mixture in ratio of 25:24:1 and filtered solution was centrifuged at 12,000× *g* for 10 min. The aqueous layer was separated and equal volume of chloroform and isoamyl alcohol in ratio of 24:1 was added, centrifuged at 12,000× *g* for 10 min. 1/10th volume of 3 M sodium acetate and 2.5 volume of absolute alcohol were added to the separated aqueous layer and stored at −20 °C overnight. Next day, this solution was centrifuged at 12,000× *g* for 10 min, the pellet was dissolved in 100 µL sterile distilled water and then absorbance was measured at 260 nm using nanodrop.

#### 4.5.2. Extracellular Protein Quantification

The Bradford method was used for quantification of extracellular proteins present in biofilm matrix. 100 µl of Bradford reagent was added to 400 µL of filtered solution (mentioned above) and incubated at room temperature for 5 min and then absorbance was measured at 595 nm using a microtiter plate reader (Multiskan Go, Thermo Fisher Scientific, Waltham, MA, USA).

#### 4.5.3. Exopolysaccharide (EPS) Quantification

EPS present in the matrix was quantified using congo red binding assay [50]. Briefly, Congo red was added to the final concentration of 40 µg/mL in 1 mL of homogenate and incubated at 37 °C for 2 h in shaking conditions. The solution was centrifuged to pellet down the cells, and the absorbance of the supernatant was measured at 490 nm. 1 mL of 0.8% saline with Congo red was used as the reference. The percentage of Congo red bound to the cells was calculated as follows: % bound Congo red = 100 – ((OD490 of test supernatant × 100)/OD OD490 of reference)) [50].

#### 4.5.4. Live Dead Assay

BacLight™ kit L7012 (Thermo Fisher Scientific, Waltham, MA, USA) was used to perform the live dead assay. 2 µL of syto9 (1:5 dilution) and PI mixture in 1:1 ratio was added to 300 µL of solubilized biofilm solution (without SDS treatment) and subjected to flow cytometry (Becton Dickinson FACS caliber, New Jersey, United States). 300 µL of solution stained individually with syto9 and PI and 300 µL of unstained culture were used as controls to eliminate the auto-fluorescence/background of the sample in flow cytometry. Double positive cells (syto9+ and PI+) contribute to the number of dead cells (total PI+). Double positive cells were considered as dead cells because PI has displaced syto9 as the membrane of these cells is damaged/compromised (dying cells) [51].

### 4.6. Resazurin Assay

In Resazurin assay, 0.015 mg/mL stock solution in 0.8% saline was used [17]. Briefly, 250 µL of 1:10 diluted culture was inoculated in a 96-well microtiter plate and incubated at 37 °C for 24 h. The unbound cells were washed off with distilled water and 20 µL of resazurin dye (HiMedia Laboratories Pvt. Ltd., Mumbai, India) diluted in 100 µL of 0.8% saline and was added in each well and incubated at 37 °C for 60 min. The fluorescence was measured at 530/590 nm excitation/emission wavelengths using the synergy HTmicroplate reader (BioTek instruments, Winooski, VT, USA).

### 4.7. Time Bound Live Dead Assay

Live dead assay was performed at 6, 18, and 24 h to study the change in the ratio of live and dead cells in weak and strong biofilm at various time points. Briefly, 25 µL of overnight grown culture (1:10 dilution of O.D at 600 nm ~ 0.3) was added to 225 µL of sterile LB in a sterile black opaque walled 96-well microtiter plate to reduce fluorescent signal crosstalk and background (Laxbro Bio-Medical Aids pvt. Ltd., India). The plate was then incubated at 37 °C for 6, 18, and 24 h. The assay was performed in triplicates. Only LB without bacterial culture was used as negative control for biofilm formation. After respective time points, the unbound cells were washed off using 0.8% normal saline and the biofilm was solubilized in 100 µL of 0.8% saline. Staining and detection of the absorbance was done as per the protocol given in the kit manual. Briefly, BacLight kit L7012 A concentrated dye solution containing equal volume of syto9 and PI (15 µL each) (Baclight kit L7012) in 5.5 mL of autoclaved molecular grade water was prepared and 100 µL of this dye solution was added in each well. Proper mixing with pipetting was done and the plate was incubated at room temperature for 15 min. After incubation, fluorescence intensity was measured using the synergy HTmicroplate reader (BioTek instruments, Winooski, VT, USA). The excitation/emission spectrum used to detect syto9 and PI stained cells was 485/530 nm and 485/630 nm, respectively. Measure of live and dead cells was calculated in term of intensity units (IU) detected of syto9 and PI. The experiment was performed in triplicates.

### 4.8. Inhibition Assays

Inhibition assay was performed to validate the role of different biofilm matrix components [36]. Briefly, 25 μL of overnight grown culture (OD at 600 nm ~ 0.3) was added to 225 μL of sterile LB in a sterile 96-well flat bottom microtiter plate (Laxbro Bio-Medical Aids pvt. Ltd., Pune, India) and incubated at 37 °C for 24 h. The assay was performed in triplicates and only LB was used as the negative control. After 24 h, the unbound cells were washed off twice with 0.8% saline and 100 μg/mL of DNase I, RNase A, and Proteinase K enzyme solution was added separately in triplicates and further incubated at 37 °C for 24 h. A set of wells containing 25 μL of overnight grown culture (OD at 600 nm ~ 0.3) and 225 µL LB only without addition of any enzyme were used as controls. After 24 h, the enzyme solution and unbound cells were washed off twice with 0.8% saline and crystal violet assay was performed to quantify the amount of biofilm. Paired *t*-test was applied to validate the decrease in biofilm formation upon enzymatic treatment statistically.

### 4.9. Addition Assay

Addition assay was performed to validate the role of different biofilm matrix components [37]. Briefly, 25 μL of overnight grown culture (OD at 600 nm ~ 0.3) was added to 225 μL of sterile LB in a sterile 96-well flat bottom microtiter plate (Laxbro Bio-Medical Aids pvt. Ltd., Pune, India). Kp cell extracted DNA and protein was added separately to the final concentration of 3 μg/mL [37] in the wells. The 96-well plate was then incubated at 37 °C for 24 h. The assay was performed in triplicates and wells containing only LB were used as the negative control. A set of wells containing 25 μL of overnight grown culture (OD at 600 nm ~ 0.3) and 225 µL LB only without addition of DNA and Protein were used as controls. After 24 h, the unbound cells were washed off twice with 0.8% saline and crystal violet assay was performed to quantify the amount of biofilm. Paired *t*-test was applied to validate the change in biofilm formation upon addition of DNA or proteins statistically.

### 4.10. Microscopy of Strong and Weak Biofilms

#### 4.10.1. CLSM

Biofilms formed by three weak and three strong isolates on glass coverslips were subjected to CLSM after 48 h. 5 mL of 1:10 diluted culture (OD at 600 nm ~ 0.3) was inoculated in a 6-well plate containing sterile glass coverslips of 22 mm diameter. The biofilm was allowed to form for 48 h at 37 °C. The unbound cells were washed off with 0.8% saline and the cells embedded in the biofilm were stained with syto9 and PI for 10 min. Excess stain was washed away with 0.8% saline and the coverslip was mounted for CLSM. Carl Ziess CLSM 780 microscope equipped with detectors and filter sets for simultaneous monitoring of Syto9 (green) (multi argon laser, 488 nm excitation, emission spectra 492–525 nm) and PI (red) (DPSS laser, 561 nm excitation, emission spectra 563-652 nm) fluorescence was used to study the arrangements of live and dead cells embedded in the biofilm matrix. Visualization of 3D structure was done using Z-stack mode of CLSM. Large section images were produced by tile scanning of strong and weak biofilms formed by all six biofilm producers. Images were analyzed using ImageJ and Zen Zeiss microscope software. The intensity units of syto9 and PI in each slice from top to bottom of the Z-stack was calculated using ImageJ for all isolates. Number of live and dead cells of all isolates was calculated from the tile images using ImageJ. Area of 1000 × 1000 pixel in tile images was used as a region of interest (ROI) to calculate the ratio of live and dead cells for all isolates.

#### 4.10.2. FEG-SEM

Biofilm was formed on 13 mm piece of silicone coated latex catheter as described above and FEG-SEM (Nova NanoSEM 450, FEI Ltd., Hillsboro, OR, USA) was performed for strong and weak biofilms formed on silicone-coated latex catheter after 24 h. SEM scanning was done at room temperature in environmental mode.

#### 4.10.3. Cell Adhesion Assay

Cell adhesion assay was performed to evaluate the adhesion ability of weak and strong biofilm producers. 5 mL of 1:10 diluted culture (OD at 600 nm ~ 0.3) was inoculated in a 6-well plate containing sterile coverslips of 22 mm diameter. Cells were allowed to adhere for 4 h at 37 °C. The unbound cells were then washed off with 0.8% saline and cells adhered on coverslip were subjected to gram staining followed by light microscopy (Magnüs MLM). The number of cells adhered on the coverslip were calculated for 10 fields at 100× magnification using ImageJ for all six isolates.

### 4.11. Statistical Analysis

All assays were performed in triplicates and standard deviation (SD) values were calculated. Nonparametric paired and unpaired student’s *t*-test were performed using Prism 8.0 Software (GraphPad, San Diego, CA, USA) to statistically evaluate the differences obtained. *p*-value of < 0.05 was considered statistically significant.

## Supplementary Materials

The following are available online at https://www.mdpi.com/2076-0817/8/4/277/s1.

## References

[1] Podschun R., Ullmann U. (1998). Klebsiella spp. as nosocomial pathogens: Epidemiology, taxonomy, typing methods, and pathogenicity factors. Clin. Microbiol. Rev..

[2] Niveditha S., Pramodhini S., Umadevi S., Kumar S., Stephen S. (2012). The isolation and the biofilm formation of uropathogens in the patients with catheter associated urinary tract infections (UTIs). J. Clin. Diagn. Res.

[3] Høiby N., Ciofu O., Johansen H.K., Song Z.J., Moser C., Jensen P.Ø., Molin S., Givskov M., Tolker-Nielsen T., Bjarnsholt T. (2011). The clinical impact of bacterial biofilms. Int. J. Oral. Sci..

[4] Geraci D.M., Bonura C., Giuffrè M., Saporito L., Graziano G., Aleo A., Fasciana T., Di Bernardo F., Stampone T., Palma D.M. (2015). Is the monoclonal spread of the ST258, KPC-3-producing clone being replaced in southern Italy by the dissemination of multiple clones of carbapenemnonsusceptible, KPC-3-producing Klebsiella pneumoniae?. Clin. Microbiol. Infect..

[5] Bonura C., Giuffrè M., Aleo A., Fasciana T., Di Bernardo F., Stampone T., Giammanco A., MDR-GNWorking Group Palma D.M., Mammina C. (2015). An Update of the Evolving Epidemic of blaKPC Carrying Klebsiella pneumoniae in Sicily, Italy, 2014: Emergence of Multiple Non-ST258 Clones. PLoS ONE.

[6] Nicolle L.E. (2014). Catheter associated urinary tract infections. Antimicrob. Resist. Infect. Control..

[7] Ong C.L.Y., Ulett G.C., Mabbett A.N., Beatson S.A., Webb R.I., Monaghan W., Nimmo G.R., Looke D.F., McEwan A.G., Schembri M.A. (2008). Identification of type-3 fimbriae in uro-pathogenic Escherichia coli reveals a role in biofilm formation. J. Bacteriol..

[8] Bandeira M., Carvalho P.A., Duarte A., Jordao L. (2014). Exploring Dangerous Connections between Klebsiella pneumoniae Biofilms and Healthcare-Associated Infections. Pathogens.

[9] Lewis K. (2008). Multidrug tolerance of biofilms and persister cells. Bacterial Biofilms.

[10] Calà C., Amodio E., Di Carlo E., Virruso R., Fasciana T., Giammanco A. (2015). Biofilm production in Staphylococcus epidermidis strains, isolated from the skin of hospitalized patients: genetic and phenotypic characteristics. New. Microbiol..

[11] Vuotto C., Longo F., Pascolini C., Donelli G., Balice M.P., Libori M.F., Tiracchia V., Salvia A., Varaldo P.E. (2017). Biofilm formation and antibiotic resistance in Klebsiella pneumoniae urinary strains. J. Appl. Microbiol..

[12] Ostria-Hernandez M.L., Juárez-de la Rosa K.C., Arzate-Barbosa P., Lara-Hernández A., Sakai F., Ibarra J.A., Castro-Escarpulli G., Vidal J.E. (2018). Nosocomial, Multidrug-Resistant Klebsiella pneumoniae Strains Isolated from Mexico City Produce Robust Biofilms on Abiotic Surfaces but Not on Human Lung Cells. Microb. Drug. Resist..

[13] De Campos P.A., Royer S., da Fonseca Batistao D.W., Araújo B.F., Queiroz L.L., de Brito C.S., Gontijo-Filho P.P., Ribas R.M. (2016). Multidrug resistance related to biofilm formation in Acinetobacter baumannii and Klebsiella pneumoniae clinical strains from different pulsotypes. Curr. Microbiol..

[14] Khodadadian R., Rahdar H.A., Javadi A., Safari M., Khorshidi A. (2018). Detection of VIM-1 and IMP-1 genes in Klebsiella pneumoniae and relationship with biofilm formation. Microb. Pathog..

[15] Schroll C., Barken K.B., Krogfelt K.A., Struve C. (2010). Role of type 1 and type 3 fimbriae in Klebsiella pneumoniae biofilm formation. BMC Microbiol..

[16] Stahlhut S.G., Struve C., Krogfelt K.A., Reisner A. (2012). Biofilm formation of Klebsiella pneumoniae on urethral catheters requires either type 1 or type 3 fimbriae. FEMS Immunol. Med. Microbiol..

[17] Peeters E., Nelis H.J., Coenye T. (2008). Comparison of multiple methods for quantification of microbial biofilms grown in microtiter plates. J. Microbiol. Methods.

[18] Diago-Navarro E., Chen L., Passet V., Burack S., Ulacia-Hernando A., Kodiyanplakkal R.P., Levi M.H., Brisse S., Kreiswirth B.N., Fries B.C. (2014). Carbapenem-resistant Klebsiella pneumoniae exhibit variability in capsular polysaccharide and capsule associated virulence traits. J. Infect. Dis..

[19] NicolauKorres A.M., Aquije G.M.D.F.V., Buss D.S., Ventura J.A., Fernandes P.M.B., Fernandes A.A.R. (2013). Comparison of biofilm and attachment mechanisms of a phytopathological and clinical isolate of Klebsiella pneumonia subsp. pneumoniae. Sci. World J..

[20] Revdiwala S., Rajdev B.M., Mulla S. (2012). Characterization of bacterial etiologic agents of biofilm formation in medical devices in critical care setup. Crit. Care Res. Pract..

[21] Anderl J.N., Zahller J., Roe F., Stewart P.S. (2003). Role of nutrient limitation and stationary-phase existence in Klebsiella pneumoniae biofilm resistance to ampicillin and ciprofloxacin. Antimicrob. Agents Chemother..

[22] Zahller J., Stewart P.S. (2002). Transmission electron microscopic study of antibiotic action on Klebsiella pneumoniae biofilm. Antimicrob. Agents Chemother..

[23] Crémet L., Corvec S., Batard E., Auger M., Lopez I., Pagniez F., Dauvergne S., Caroff N. (2013). Comparison of three methods to study biofilm formation by clinical strains of Escherichia coli. Diagn. Microbiol. Infect. Dis..

[24] Balestrino D., Ghigo J.M., Charbonnel N., Haagensen J.A., Forestier C. (2008). The characterization of functions involved in the establishment and maturation of Klebsiella pneumoniae in vitro biofilm reveals dual roles for surface exopolysaccharides. Environ. Microbiol..

[25] Feneley R.C., Hopley I.B., Wells P.N. (2015). Urinary catheters: History, current status, adverse events and research agenda. J. Med. Eng. Technol.

[26] Donlan R.M. (2001). Biofilms and device-associated infections. Emerg. Infect. Dis..

[27] Tunney M.M., Jones D.S., Gorman S.P. (1999). Biofilm and biofilm-related encrustation of urinary tract devices. Methods Enzymol..

[28] Lee K.H., Park S.J., Choi S., Uh Y., Park J.Y., Han K.H. (2017). The influence of urinary catheter materials on forming biofilms of microorganisms. J. Bacteriol. Virol..

[29] Bandeira M., Borges V., Gomes J.P., Duarte A., Jordao L. (2017). Insights on Klebsiella pneumoniae biofilms assembled on Different Surfaces using phenotypic and genotypic approaches. Microorganisms.

[30] Hu W., Li L., Sharma S., Wang J., McHardy I., Lux R., Yang Z., He X., Gimzewski J.K., Li Y. (2012). DNA builds and strengthens the extracellular matrix in Myxococcus xanthus biofilms by interacting with exopolysaccharides. PLoS ONE.

[31] Huseby M.J., Kruse A.C., Digre J., Kohler P.L., Vocke J.A., Mann E.E., Bayles K.W., Bohach G.A., Schlievert P.M., Ohlendorf D.H. (2010). Beta toxin catalyzes formation of nucleoprotein matrix in staphylococcal biofilms. Proc. Natl. Acad. Sci. USA.

[32] Domenech M., García E., Prieto A., Moscoso M. (2013). Insight into the composition of the intercellular matrix of Streptococcus pneumoniae biofilms. Environ. Microbiol..

[33] Okshevsky M., Meyer R.L. (2015). The role of extracellular DNA in the establishment, maintenance and perpetuation of bacterial biofilms. Crit. Rev. Microbiol..

[34] Flemming H.C., Wingender J. (2010). The biofilm matrix. Nat. Rev. Microbiol..

[35] Jakubovics N.S., Shields R.C., Rajarajan N., Burgess J.G. (2013). Life after death: The critical role of extracellular DNA in microbial biofilms. Lett. Appl Microbiol..

[36] Tetz G.V., Artemenko N.K., Tetz V.V. (2009). Effect of DNase and antibiotics on biofilm characteristics. Antimicrob. Agents Chemother..

[37] Harmsen M., Lappann M., Knøchel S., Molin S. (2010). Role of extracellular DNA during biofilm formation by listeria monocytogenes. Appl. Environ. Microbiol..

[38] Bayles K.W. (2007). The biological role of death and lysis in biofilm development. Nat. Rev. Microbiol..

[39] Lewis K. (2009). Programmed death in bacteria. Microbiol. Mol. Biol. Rev..

[40] Claverys J.P., Håvarstein L.S. (2007). Cannibalism and fratricide: Mechanisms and raisons d’etre. Nat. Rev. Microbiol..

[41] Renelli M., Matias V., Lo R.Y., Beveridge T.J. (2004). DNA-containing membrane vesicles of Pseudomonas aeruginosa PAO1 and their genetic transformation potential. Microbiology.

[42] Webb J.S., Thompson L.S., James S., Charlton T., Tolker-Nielsen T., Koch B., Givskov M.K., Jelleberg S. (2003). Cell death in Pseudomonas aeruginosa biofilm development. J. Bacteriol..

[43] Salgado-Pabón W., Du Y., Hackett K.T., Lyons K.M., Arvidson C.G., Dillard J.P. (2010). Increased expression of the type IV secretion system in piliated Neisseria gonorrhoeae variants. J. Bacteriol..

[44] Fagerlind M.G., Webb J.S., Barraud N., McDougald D., Jansson A., Nilsson P., Harlén M., Kjelleberg S., Rice S.A. (2012). Dynamic modelling of cell death during biofilm development. J. Theor. Biol..

[45] Paczosa M.K., Mecsas J. (2016). Klebsiella pneumoniae: Going on the offense with a strong defense. Microbiol. Mol. Biol. Rev..

[46] Singla S., Harjai K., Chhibber S. (2014). Artificial Klebsiella pneumoniae biofilm model mimicking in vivo system: altered morphological characteristics and antibiotic resistance. J. Antibiot..

[47] Stepanović S., Ćirković I., Ranin L., SvabićVlahović M. (2004). Biofilm formation by Salmonella spp. And Listeria monocytogenes on plastic surface. Lett. Appl. Microbiol..

[48] Shmaefsky B.R. (1990). Artificial urine for laboratory testing. Am. Biol. Teach..

[49] Wu J., Xi C. (2009). Evaluation of different methods for extracting extracellular DNA from biofilm matrix. Appl. Environ. Microbiol..

[50] Madsen J.S., Lin Y.C., Squyres G.R., Price-Whelan A., de Santiago Torio A., Song A., Cornell W.C., Sørensen S.J., Xavier J.B., Dietrich L.E. (2015). Facultative control of matrix production optimizes competitive fitness in Pseudomonas aeruginosa PA14 biofilm models. Appl. Environ. Microbiol..

[51] Accurate Assessment of Microbial Viability by Flow Cytometry 2011, July. BioProbes 65. https://www.thermofisher.com/in/en/home/references/newsletters-and-journals/bioprobes-journal-of-cell-biology-applications/bioprobes-issues-2011/bioprobes-65-july-2011/live-dead-baclight-meets-the-attune-acoustic-focusing-cytometer.html.

